# *Atractylodes macrocephala* III suppresses EMT in cervical cancer by regulating IGF2BP3 through ETV5

**DOI:** 10.1111/jcmm.18081

**Published:** 2024-02-15

**Authors:** Meixia Wang, Jingwen Meng, Hongyun Wang, Huijuan Hu, Ying Hong

**Affiliations:** ^1^ Department of Gynecology and Obstetrics Nanjing Drum Tower Hospital, Clinical College of Nanjing University of Chinese Medicine Nanjing China; ^2^ Department of Gynecology and Obstetrics Wenzhou Hospital of Integrated Traditional Chinese and Western Medicine Wenzhou China; ^3^ Department of Gynecology and Obstetrics Nanjing Drum Tower Hospital, The Affiliated Hospital of Nanjing Medical University Nanjing China; ^4^ Department of Gynecology and Obstetrics Nanjing Drum Tower Hospital, The Affiliated Hospital of Nanjing University Medical School Nanjing China

**Keywords:** atractylodes macrocephala III, cervical cancer, EMT, ETV5, IGF2BP3

## Abstract

*Atractylodes macrocephala* III (ATL III), with anti‐inflammatory and antitumor effects, is the main compound of *Atractylodes macrocephala*. Whether ATL III has an effect on cervical cancer and the specific mechanism are still unclear. Here, we investigated the effects of ATL III on cervical cancer cells at different concentrations and found that ATL III downregulates insulin‐like growth factor 2 mRNA‐binding protein 3 (IGF2BP3), which was found to be highly expressed in cervical cancer tissue by RNA‐Seq. In this study, we found that ATL III promotes apoptosis and regulates epithelial–mesenchymal transition (EMT) in cervical cancer cells (HeLa and SiHa cells) and that IGF2BP3 is a common target gene of ATL III in HeLa and SiHa cells. The expression level of IGF2BP3 in cervical cancer cells was proportional to their migration and invasion abilities. This was verified by transfection of cells with a small interfering RNA and an IGF2BP3 overexpression plasmid. After ATL III treatment, the migration and invasion abilities of cervical cancer cells were obviously reduced, but these effects were attenuated after overexpression of IGF2BP3. In addition, the transcription factor IGF2BP3 was predicted by the JASPAR system. After intersection with our sequencing results, we verified the promotional effect of ETV5 (ETS translocation variant 5) on IGF2BP3 and found that ALT III inhibited ETV5. In general, our research showed that ATL III inhibits the migration and invasion of cervical cancer cells by regulating IGF2BP3 through ETV5.

## 1 INTRODUCTION

Cervical cancer ranks fourth among female tumours in terms of morbidity and mortality rates.^1^ Globally, an average of 300,000 women die of cervical cancer each year.^2^ Persistent infection with high‐risk HPV causes approximately 90% of deaths related to cervical cancer. Although regular cervical cancer screening and the promotion and popularization of HPV vaccines have reduced the incidence of cervical cancer, it remains one of the leading causes of death among women of childbearing age in low‐income countries. Currently, cervical cancer treatment mainly includes surgery, chemotherapy, radiation, or a combination; however, standard treatment has caused great damage to the mental and physical health of female patients, and the recurrence and metastasis of tumours is still a problem that needs to be solved.^3^ Approximately 30%–40% of cervical cancer cases reoccur.^4^ Several factors are responsible for the development, progression and invasion of cervical cancer, including persistent HPV infection (especially HPV 16 and 18). In light of the lack of effective and safe treatment strategies for cervical cancer resulting from HPV infection, the development of sensitive detection methods and the exploration of new treatment strategies can actively contribute to eliminating cervical cancer.

Several RNA‐binding proteins and RNAs are reported to play essential roles in the regulation of posttranscriptional translation and the regulation of tumour development. IGF2BP3 (insulin‐like growth factor 2 mRNA‐binding protein 3) is a member of the insulin‐like growth factor 2‐mRNA binding protein family, a 69 kDa protein‐coding gene located on chromosome 7p15.3.^5^ In numerous tumour‐related studies, scholars have found that IGF2BP3 promotes the occurrence and development of tumours. It has been shown that phenotypic attachment morphology and cell–cell adhesion are more prominent in tumour cells overexpressing IGF2BP3 in vitro than in cells with low expression of IGF2BP3^6^; in vivo, overexpression of IGF2BP3 can increase tumour cell formation and metastasis.^7^ Researchers have shown that IGF2BP3 prevents miRNA‐mediated degradation of the Snail and Slug transcription factors in triple‐negative breast cancer in vivo,^8^ and the stem cell factor SOX2 is transcriptionally regulated by Snail. The level of IGF2BP3 in hepatocellular carcinoma tumour‐initiating stem cells was higher than that in all tumour cells.^9^ The physical binding of IGF2BP3 and hsa_circ_ 0003258 activates ERK‐related signalling pathways, triggering EMT processes and ultimately accelerating the progression of prostate cancer.^10^ An in vitro study of nasopharyngeal carcinoma showed that IGF2BP3 regulates key EMT regulatory factors by activating the AKT/mTOR signalling pathway, thus promoting the migration and invasion of nasopharyngeal carcinoma cells.^11^ In vivo, IGF2BP3 expression is significantly increased in cervical intraepithelial neoplasia (CIN) Type III lesions and squamous cell carcinoma (SCC) compared to normal cervical tissue.^12^, ^13^ In multiple studies, IGF2BP3 levels are expected to be used to assess the progression of cervical lesions and predict the prognosis of SCC. However, the mechanism by which IGF2BP3 regulates cervical cancer remains unclear.

*Atractylodes macrocephala* Koidz is a common Chinese herbal medicine that invigorates the spleen and dries dampness. There are many diseases that can be treated with *Atractylodes macrocephala* Koidz, including gastrointestinal disorders, cancer, osteoporosis, Alzheimer's disease and female cervical problems. *Atractylodes* is also commonly used to treat cervical cancer. The main chemical component of *Atractylodes macrocephala*, sesquiterpenoid compound atractyloside III (ATL III), has been reported to increase the function of lymphocyte immunity.^14^ ATL‐III inhibited the growth of A549 cells (human lung cancer cell line), increased the release of lactate dehydrogenase, regulated the activation of caspase‐3 and caspase‐9 induced by the cell cycle and regulated mitochondrial function to split PAPR.^15^ However, whether ATL III has an effect on cervical cancer and the specific mechanism are still unclear.

Due to the lack of available literature regarding the effect of ATL III on cervical cancer, the purpose of this study was to investigate the effect and mechanism of ATL III on cervical cancer. The differential gene was obtained by identifying RNA‐Seq in clinical tissues of cervical cancer, and IGF2BP3 was identified as the common target gene for ATL III to act on HeLa and SiHa cells. In addition, the biological functions of ATL III in cervical cancer, including cell vitality, apoptosis, migration and invasion, were studied in vitro. Finally, we explored the possible role of ETV5 as a transcription factor in the role of ATL III in cervical cancer via IGF2BP3. This study suggests that ATL III may provide a new drug therapy for the regulation of cervical cancer migration and invasion.

## 2 MATERIALS AND METHODS

### 2.1 Cell lines and chemical reagents

We obtained cell lines (HeLa with HPV 18+ and SiHa with HPV 16+) from the Shanghai Cell Bank of the Chinese Academy of Sciences. The cells were cultured in a humidified incubator at 37°C and 5% CO2 in Dulbecco's modified Eagle's medium (DMEM) containing 10% fetal bovine serum (FBS, Gibco) and 1% penicillin–streptomycin (Thermo Scientific). ATL III was purchased from MedChemExpress (purity ≥99%). The ATL III reserve solution of 10 mM/L was prepared in dimethyl sulfoxide (DMSO); in all, DMSO concentrations were less than 0.1%. Primary antibodies against IGF2BP3 (177477), E‐cadherin (231303), N‐cadherin (76011), Slug (302780) and Snail (216347) were purchased from Abcam, ETV5 (sc‐100941) was purchased from Santa Cruz and those against vimentin (10366‐1‐AP) and β‐actin (81115‐1‐RR) as well as secondary antibody (SA00001‐2) were purchased from Abcam. Annexin V‐FITC/PI apoptosis detection kits and CCK‐8 assay kits were purchased from Beyotime. All reagents were used according to the instructions.

### 2.2 Patients and tissue specimens

Tissue samples were collected from Nanjing Drum Tower Hospital. The Ethics Committee of Nanjing Gulou Hospital approved the study (2019‐138‐01). Four pairs of fresh normal cervical tissue samples and stage I b cervical squamous cell carcinoma samples with HPV16+ (from January 2022 to June 2022) were collected (stored at −80°C) for RNA‐Seq. Twelve pairs of paraffin‐embedded cervical samples (including normal (*n* = 12), CIN I (*n* = 12), CIN III (*n* = 12), cervical cancer stage Ib (*n* = 12) and paracancerous tissue (*n* = 12) samples) were collected for immunohistochemical analysis. All patients provided written informed consent prior to sample collection.

Specific case information can be found in the supplementary materials (Table S1).

### 2.3 RNA sequencing (RNA‐Seq) data analysis

The collected samples were analysed by transcriptomics based on the Illumina sequencing technology platform. RNA‐Seq was performed by eke Biotech (www.ekebio.com). In brief, total RNA was extracted using TRIzol Reagent, purified RNA was quantified using an ND‐2000 spectrophotometer and RNA integrity was detected using the Agilent 2100/Lab Chip GX. RNA‐Seq libraries were then prepared with Agilent’ s Sure Select Strand‐Specific RNA Library Preparation Kit, followed by AMPure XP Bead size selection. The sequence was directly determined with PE150 mode sequencing using the Illumina NovaSeg 6000 sequencing platform. All selected patients did not undergo radiotherapy, chemotherapy or other treatment before enrolment.

### 2.4 Bioinformatics differential gene analysis

Differentially expressed genes (DEGs) were screened by bioinformatics analysis of RNA‐Seq data with the thresholds of |Log_2_FC| ≥ 1 and *p* < 0.05. The biological function was analysed from the aspects of biological process, cell component and molecular function categories by GO analysis. KEGG pathway analysis was used to identify enriched biological pathways. Enrichment analysis was performed by using the R software package clusterProfiler (version 3.14.3). *p* < 0.05 and FDR < 1 were considered statistically significant. Then, a volcano map and heatmap were constructed online (http://www.sangerbox.com/tool).

### 2.5 Cell viability assay

For the logarithmic growth stage, HeLa and SiHa cells were uniformly inoculated into 96‐well plates at 100 μL (3000 cells) per well. Six hours later, the cells were incubated with ATL III (0, 1, 10, 100, 200, and 500 μM) for 24 hours. On the second day, CCK‐8 solution was added to the medium by drops (10 μL/well). The samples were incubated at 37°C for 2–4 h (the specific time was estimated by naked eye observation), and the absorbance at 450 nm was measured with an enzyme label instrument.

### 2.6 Transwell assay

Transwell chambers containing Matrigel were purchased from Corning. Two hundred microliters of serum‐free cell suspension (density ~5 × 10^3^ cells/well) with or without 200 μM ATL III was inoculated in the upper compartment. Then, 600 μL complete medium was added to the lower compartment and slightly immersed in the upper compartment. After incubation for 24 h, the cells were fixed with 4% paraformaldehyde for 15 min, gently scraped off the nonmetastases and stained with 0.5% crystal violet. Five fields were randomly selected under the microscope, and the cells were counted.

### 2.7 Wound‐healing assay and colony formation experiment

Cells were evenly inoculated in a 6‐well plate (~1 × 10^6^ cells/well). A pipette tip (10 μL) was used to make artificial wounds by scratching after the cells reached 80% confluence in a monolayer and then rinsed with PBS at room temperature to remove debris and isolated cells. The images at 0 h and 24 h were obtained by inverted microscopy after treatment with or without 200 μM ATL III in serum‐free DMEM. The wound area was detected using ImageJ software (version 1.51j8). The calculation formula for mobility was (initial area–24 h wound area)/initial area. Appropriate numbers of cells were plated in a 6‐well plate (~1 × 10^3^ cells/well). The cells were then treated with medium with or without ATL III for 7 days, and when colony formation was observed, the medium was discarded. The colonies were fixed with formaldehyde, stained with crystal violet, photographed and counted with a camera.

### 2.8 Flow cytometry

Cells were treated with or without ATL III (200 μM) for 24 h. After digestion, cells were collected by centrifugation (including cells in cell medium), gently washed with precooled PBS and centrifuged again. According to the instructions of the apoptosis detection kit, 195 μL of binding solution was added to each sample, and the resuspended cells were gently pipetted up and down. When there were no clumps of cells in the cell suspension, the staining solution was added, and the cell suspension was gently blown again. After incubation at 4°C for 20 min under dark conditions, analysis was performed using BD's FACS C6 flow cytometer, and the data were analysed using FlowJo software (version 10.8.1).

### 2.9 Immunohistochemical experiment

The paraffin‐embedded tissue was sectioned continuously with a thickness of 4 μm for immunohistochemistry (IHC). After being baked, dewaxed with xylene and hydrated with ethanol, the endogenous peroxidase active sites were blocked, and the slices were cooked in an antigen‐repair solution and soaked with 3% hydrogen peroxide. After blocking with goat serum at room temperature for 30 mins, the slices were incubated with anti‐IGF2BP3 (1:200, Abcam). Following incubation with the secondary antibody (1:5000), haematoxylin was applied to stain the nucleus, and then routine dehydration and blocking were performed. Immunohistochemistry images were obtained by optical microscopy.

### 2.10 Western blot

Cells were collected after the corresponding treatment, protein was extracted using strong RIPA lysis buffer (Beyotime), and a bicinchoninic acid assay was used to quantify protein concentrations. After heating and denaturation, electrophoresis was performed on SDS–PAGE gels, and the proteins were then transferred to PVDF membranes using wet transfer. The membrane was sealed with 5% skim milk (dissolved in TBST) at room temperature for 2 h. Primary antibodies against IGF2BP3 (1:4000), E‐cadherin (1:5000), N‐cadherin (1:1000), Slug (1:1000) and Snail (1:1000) were incubated with the membrane at 4°C overnight. After washing with TBST three times, the secondary antibody (1:5000) was dissolved in the blocking solution, and the membrane was incubated at room temperature for 2 h. Electrophoresis gel imaging analysis was used to detect the protein using enhanced chemiluminescence reagents (Sangon). The relative expression was analysed using ImageJ software.

### 2.11 RNA extraction and RT–PCR analysis

Then, 500 μL TRIzol reagent was added to each well of the 6‐well plate (80 mg tissue into 1 mL TRIzol reagent). After grinding, it was incubated at 15–30°C for 15 min to fully crack. Centrifuge at 4°C at 12,000 rpm for 5 min, and discard the precipitate. Chloroform was added according to 200 μL chloroform/ml TRIzol, violently shaken for 15 s and incubated at 15–30°C for 2–3 min. Centrifuge at 4°C at 12,000 RPM for 15 min. The upper water phase was sucked into anot her centrifuge tube. Isopropyl alcohol was added to an equal volume, shaken slightly and incubated at room temperature for 10 min. At 4°C and 12,000 × g, the sample was centrifuged for 10 min, the supernatant was discarded and the RNA in the bottom of the tube was removed. Add 1 mL of 75% ethanol, swirl well, and suspend precipitation. Centrifuge at 7500 rpm for 5 min at 4°C, discard the supernatant as much as possible. Dry at room temperature or under vacuum for 5–10 min. DEPC dissolved RNA precipitation in 30 μL of water and measured the OD value after 60°C water bathing for 20 min to quantify the RNA concentration. The PrimeScriptTM RT reagent kit (Takara) was used to generate cDNA from total RNA (a 10 μL‐reaction system containing 1000 ng total RNA). The experiment was carried out by the SYBR Green method. All steps refer to the reagent specifications. Specific information on the sequences of primers used in the experiment is listed in the supplementary Table S2. 18S or GAPDH was used as an internal control.

### 2.12 Cell transfection

The pcDNA3.1‐IGF2BP3 and short hairpin psi‐H1‐shIGF2BP3 plasmids were commercially constructed by Hanbio, Shanghai. The pcDNA3.1 and psi‐H1 empty vectors were used as controls. HeLa and SiHa cells were seeded in 6‐well plates and transfected in serum‐free medium by Lipofectamine 3000 (Invitrogen, Thermo Fisher Science, Inc.). After 6 h of transfection with pcDNA3.1‐IGF2BP3 and psi‐H1‐shIGF2BP3, the transfection efficiency was analysed by western blotting. The cells were transferred to complete medium containing FBS and incubated continuously for 48 hours, and cells with stable transfection efficiency were used for subsequent processing.

### 2.13 Immunofluorescence analysis

The cells were inoculated at a density of 1 × 10^5^ cells/mL in 6‐well plates with a slide, and the cells were cultured with serum with or without ATL III for 24 h. Then, the cells were washed with cold PBS three times, fixed with 4% paraformaldehyde for 30 min and sealed with goat serum for 30 min. After incubation for 12 h with an anti‐IGF2BP3 diluent (1:200), the cells were treated with the fluorescent secondary antibody (1:500) labelled with fluorescein isothiocyanate (FITC) for 1 h. The cells were washed three times with PBS and dyed with 100 ng/mL DAPI for 10 min. Fluorescence microscopy was used to observe and photograph the cells.

### 2.14 Statistical analysis

Statistical analysis was performed using GraphPad Prism software (version 9.0). All quantitative results are presented as the means with standard errors. Multiple comparisons were carried out using one‐way ANOVA with Bonferroni correction for normally distributed data, and two groups were compared using an unpaired *t*‐test for normally distributed data. Statistical significance was determined by a *p* value of 0.05.

## 3 RESULTS

### 3.1 ATL III inhibits the proliferation of cervical carcinoma cells in vitro

**FIGURE 1:**
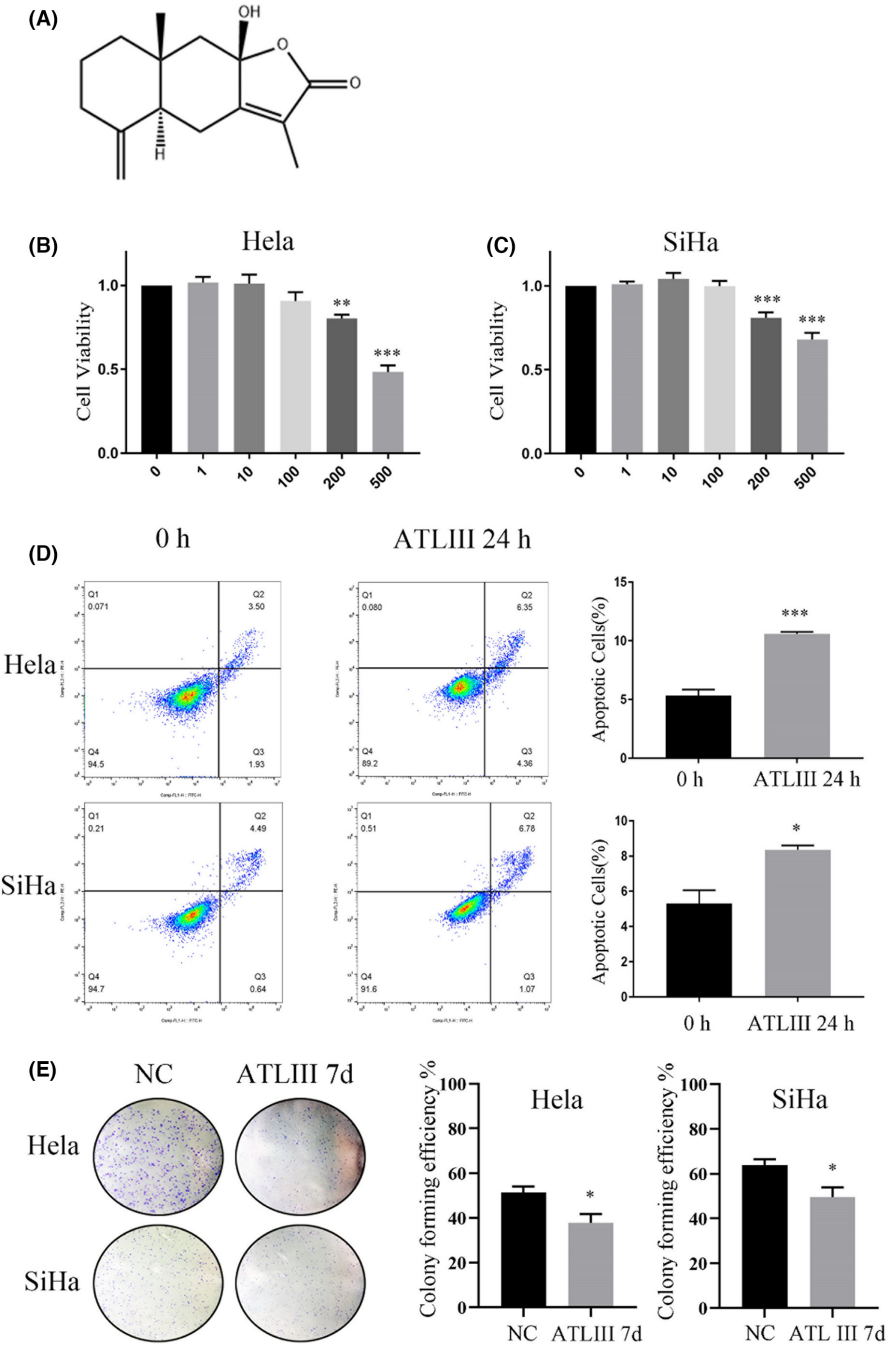
ATL III inhibits the proliferation of cervical carcinoma cells in vitro. (A) The chemical molecular structure of ATL III. (B and C) CCK‐8 assay of ATL III at different concentrations in HeLa and SiHa cells. (D) Apoptosis detection of HeLa and SiHa cells treated with 200 μM ATL III for 24 h. (E) Colony formation assay in HeLa and SiHa cells treated with the concentrations in D for 7 days. Data are presented as the mean ± SEM. *N* = 3, *p < 0.05, **p < 0.01, ***p < 0.001. No marker means no significant difference.

ATL III is the main active ingredient of *Atractylodes macrocephala*, and its structural formula is shown in Figure 1A. With CCK‐8 analysis, different concentrations of ATL III were used to treat cervical cancer cells to determine the effects of ATL III on HeLa and SiHa cells. In the CCK‐8 assay, ATL III significantly inhibited the growth of both HeLa and SiHa cells, and SiHa cells were more sensitive to ATL III (Figure 1B,C) at the IC50 concentration of ATL III. Afterward, we performed in vitro experiments using 200 μM ATL III in different analyses. According to flow cytometry, ATL III promotes the apoptosis of cervical cancer cells and is more apparent in HeLa cells (Figure 1D). Cloning experiments showed that ATL III obviously inhibited the formation of tumour spheres in HeLa and SiHa cells (Figure 1E). In general, ATL III showed strong inhibition of cervical cancer cell aggressiveness.

### 3.2 IGF2BP3 is highly expressed in cervical cancer patients in the clinic

By analysing the RNA‐Seq detection of normal cervical tissue and cervical cancer tissue samples, 34,859 differentially expressed genes were identified in normal cervical and cervical cancer tissue. A total of 962 differentially expressed genes were obtained with the level of gene‐specific expression (|Log2FC| ≥ 1, *p* < 0.05). KEGG enrichment analysis showed that DEGs were largely concentrated in virus infection, pathways in cancer, neuroactive ligand–receptor interaction, the camp signalling pathway and the PI3K‐Akt signalling pathway. In total, 377 genes were upregulated. IGF2BP3 was one of the most significantly upregulated genes.

### 3.3 ATL III inhibits IGF2BP3 expression in cervical cancer cells

**FIGURE 2:**
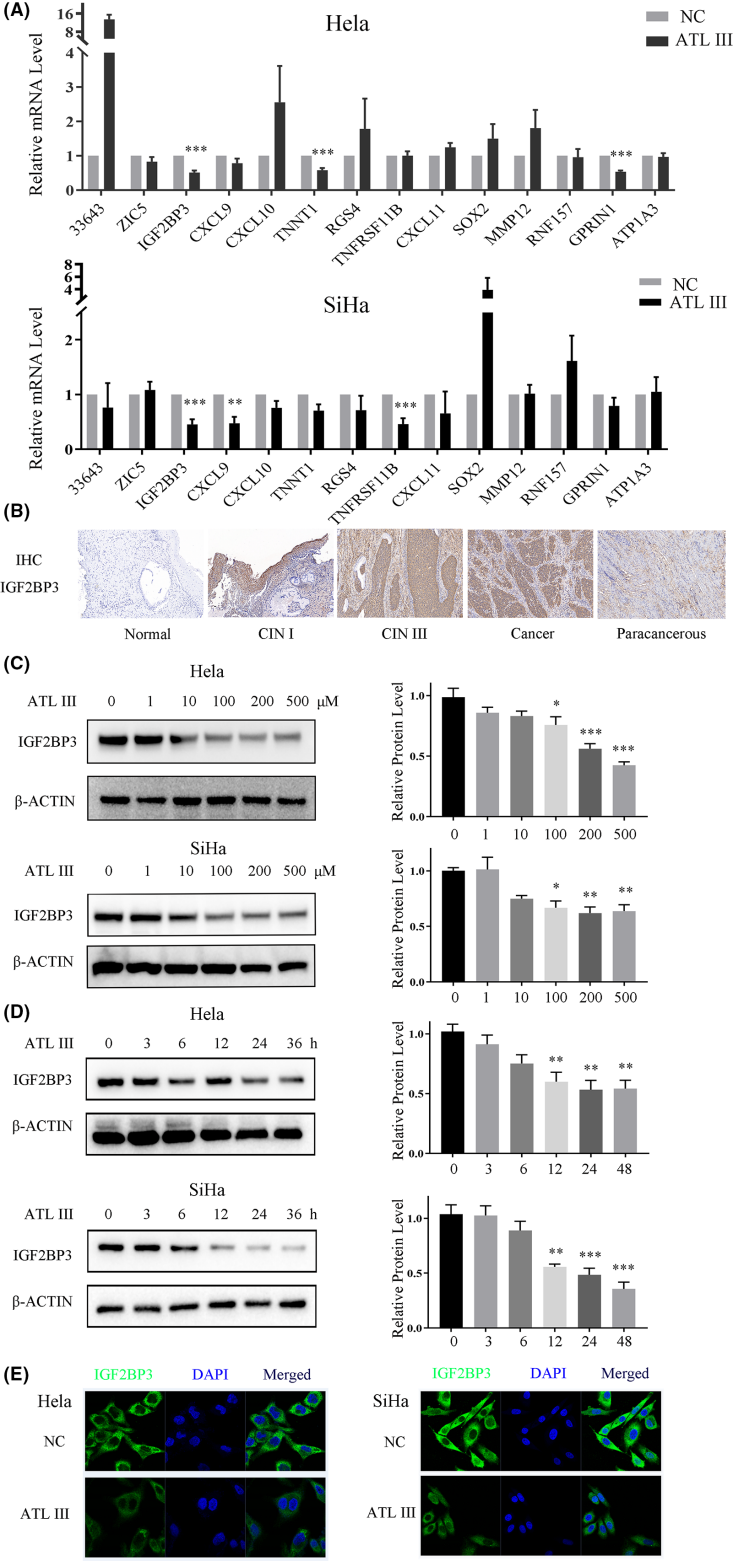
ATL III inhibits IGF2BP3 expression in HeLa and SiHa cells. (A) qRT–PCR showed the top 14 gene changes in DEGs after ATL III treatment in HeLa and SiHa cells. (B) IHC showed IGF2BP3 expression in normal cervical tissue, CIN I, CIN III, cervical cancer and paracancerous tissues. (C and D) Western blot showing the protein levels in HeLa and SiHa cells after ATL III treatment at different concentrations and times. (E) IF showing that IGF2BP3 was localized in the cytoplasm and that its expression was decreased after ATL treatment. Data are presented as the mean ± SEM. *N* = 3. *p < 0.05, **p < 0.01, ***p < 0.001. No marker means no significant difference.

The top 14 differentially expressed genes of mRNA‐Seq were used for further research. After the treatment of cervical cancer cells with ATL III, qRT–PCR results showed that both groups of cell lines showed significant reductions in the transcription levels of IGF2BP3 after drug intervention (Figure 2A). The results of IHC revealed that the expression of IGF2BP3 protein in CIN III and cervical carcinoma tissues was increased compared to that in normal tissues (Figure 2B). The WB experiments showed that the protein levels of IGF2BP3 were significantly inhibited in both HeLa and SiHa cells after ATL III intervention in a concentration‐dependent manner (Figure 2C). The protein expression level of IGF2BP3 decreased at an ATL III concentration of 100 μM, and the expression of IGF2BP3 in HeLa cells decreased more significantly at an ATL III concentration of 200 μM. The effect of ATL III cells on the protein expression of IGF2BP3 was also time‐dependent (Figure 2D). The protein expression of IGF2BP3 decreased significantly after 12 hours of intervention, and the decrease was more significant in SiHa cells than in HeLa cells. Immunofluorescence (IF) experiments showed that the IGF2BP3 protein was located in the cytoplasm and was significantly downregulated after ATL III intervention (Figure 2D).

### 3.4 ATL III inhibits the migration and invasion of cervical cancer cells

**FIGURE 3:**
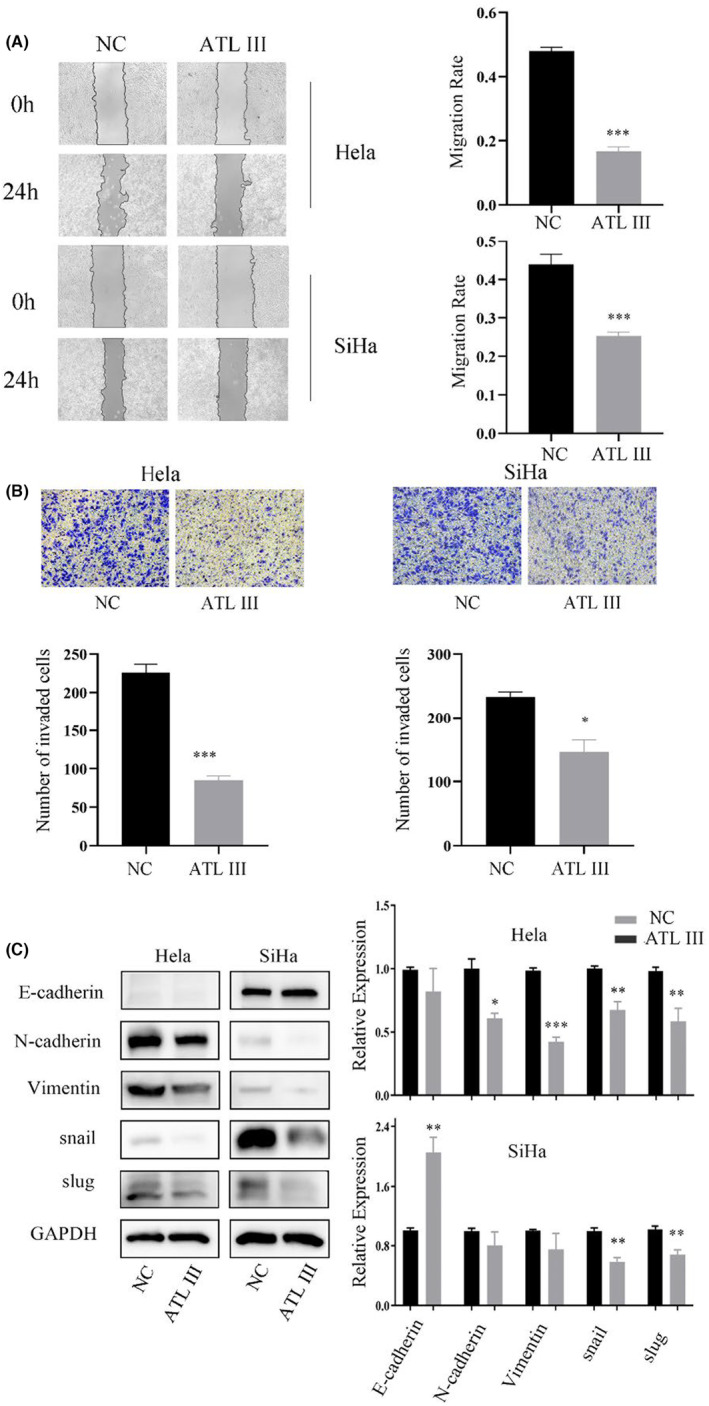
ATL III inhibits the metastasis of HeLa and SiHa cells. (A) Wound‐healing assay showing the effect of ATL III on HeLa and SiHa cell migration. (B) Transwell assays showed that ATL III inhibited the invasion of cervical cancer cells. (C) Western blot showing the protein levels of E‐cadherin, N‐cadherin, vimentin, Snail and Slug; β‐Actin was used as the loading control. Data are presented as the mean ± SEM. *N* = 3. *p < 0.05, **p < 0.01, ***p < 0.001. No marker means no significant difference.

To investigate the mechanism by which ATL III affects cervical cancer cells, migration and invasion experiments were performed after ATL III treatment, and the wound‐healing experiment showed that the wound‐healing ability of HeLa and SiHa cells decreased significantly after ATL III treatment (Figure 3A). The results of the invasion experiment showed that the invasion ability of cells in both groups decreased after drug intervention, and invasion was more significantly inhibited in HeLa cells than in SiHa cells (Figure 3B). The results showed that ATL III inhibited the migration and invasion of cervical cancer cells. Then, we detected EMT biomarker proteins. As ATL III inhibits the migration and invasion of cervical cancer cells, we assessed whether ATL III can regulate EMT in cervical cancer cells. The results of the WB experiment showed that N‐cadherin and vimentin levels in HeLa cells were significantly decreased, E‐cadherin levels in SiHa cells were significantly increased, and Snail and Slug levels in the two groups of cells were significantly decreased after drug intervention (Figure 3C). Thus, ATL III regulates EMT in cervical cancer cells.

### 3.5 IGF2BP3 can regulate EMT in cervical cancer cells

**FIGURE 4:**
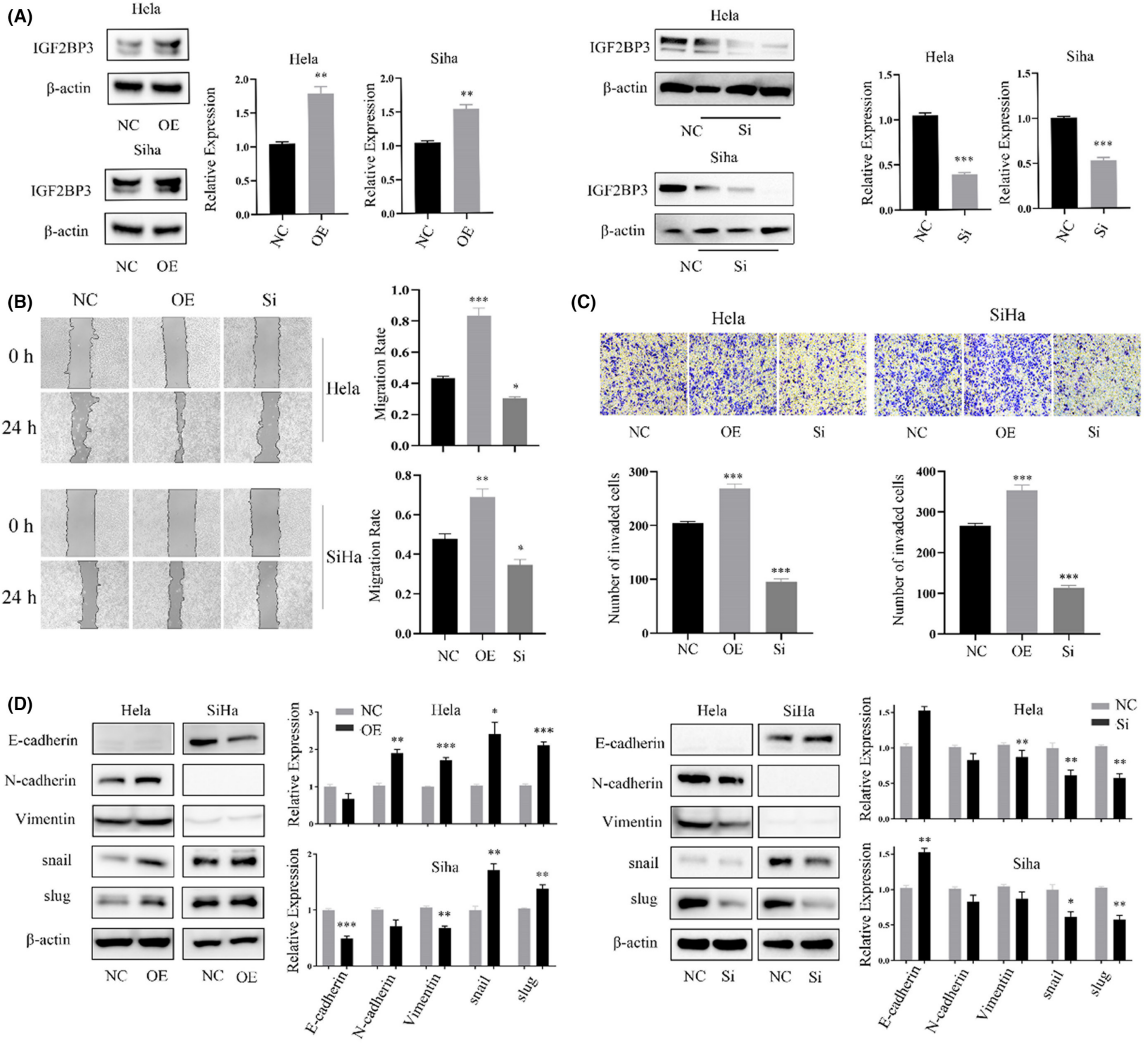
IGF2BP3 promotes the metastasis of HeLa and SiHa cells. (A) WB showed the protein expression level after IGF2BP3 transfection. (B) The wound healing assay showed the migration ability after IGF2BP3 transfection. (C) Transwell assays showed the invasion ability after IGF2BP3 transfection. (D) WB experiments showed the EMT protein level after transfection of IGF2BP3. Data are presented as the mean ± SEM. *p < 0.05, **p < 0.01, ***p < 0.001. *N* = 3. No marker means no significant difference.

To explore the mechanism of IGF2BP3 in cervical cancer cells, we overexpressed or silenced IGF2BP3 in cervical cancer cells, and WB experiments were used to verify the expression of EMT biomarker proteins after transfection (Figure 4A). The wound‐healing assay showed that overexpression of IGF2BP3 significantly promoted the migration ability of cervical cancer cells, whereas silencing IGF2BP3 inhibited the migration ability of cervical cancer cells compared with that in the control group (Figure 4B). In addition, transwell experiments showed that cervical cancer cells overexpressing IGF2BP3 showed stronger invasion ability than control cells (Figure 4C). In summary, our research showed that IGF2BP3 is involved in the migration and invasion of cervical cancer cells in vitro. The results showed that vimentin and N‐cadherin were highly expressed in HeLa cells, and E‐cadherin was almost not expressed, suggesting that HeLa cells present mesenchymal characteristics; E‐cadherin was highly expressed in SiHa cells, and N‐cadherin was almost not expressed, suggesting that SiHa cells present epithelial characteristics. Snail and Slug were expressed in both groups of cells. After plasmid transfection, the E‐cadherin level was significantly decreased in SiHa cells with IGF2BP3 overexpression, while E‐cadherin and vimentin levels were increased in HeLa cells with IGF2BP3 overexpression versus the control cells; Snail and Slug levels were increased in both groups. After knocking out IGF2BP3, N‐cadherin and vimentin levels were significantly decreased in HeLa cells, E‐cadherin levels were significantly decreased in SiHa cells, and Snail and Slug levels were decreased in both groups. These results show that IGF2BP3 can promote the EMT of cervical cancer cells by regulating N‐cadherin, E‐cadherin, vimentin, Snail and Slug expression (Figure 4D).

### 3.6 ATL III regulates EMT through IGF2BP3 in cervical cancer cells

**FIGURE 5:**
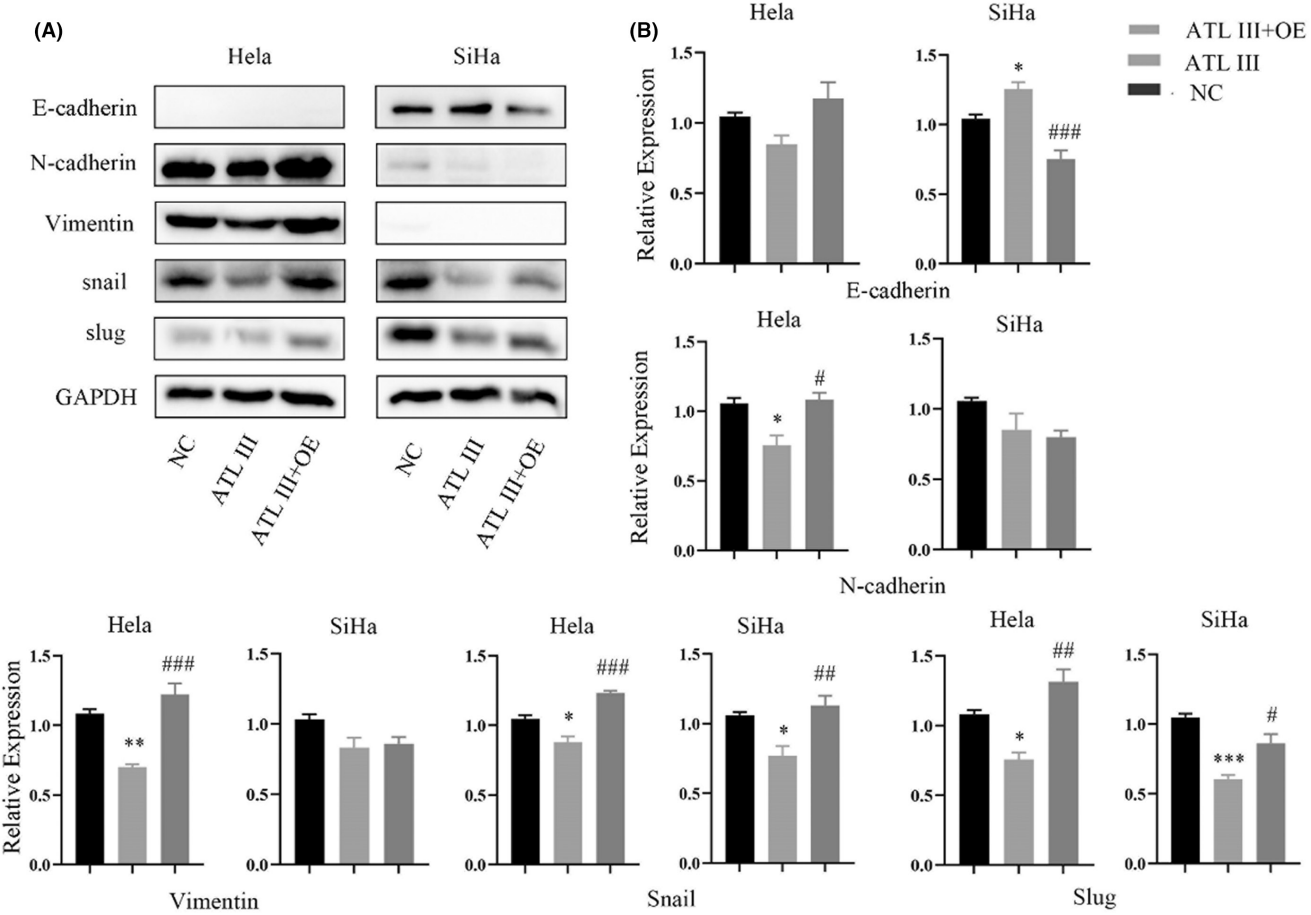
ATL III regulates EMT through IGF2BP3 in cervical cancer cells. (A, B) WB assay showing the level of EMT proteins in HeLa and SiHa cells overexpressing IGF2BP3 after ATL treatment. Data are presented as the mean ± SEM. *N* = 3. *Compared with NC *p* < 0.05, **compared with NC *p* < 0.01, ***compared with NC *p* < 0.001. #compared with ALT III *p* < 0.05, ^##^compared with ALT III *p* < 0.01, ^###^compared with ALT III *p* < 0.001. No marker means no significant difference.

After the treatment of HeLa and SiHa cells with ATL III, the expression of IGF2BP3 and EMT‐related biomarkers, such as N‐cadherin, vimentin, Slug and Snail, decreased significantly (Figure 5A,B); however, the overexpression of IGF2BP3 reversed the protein expression changes in the above genes. The expression of E‐cadherin increased after treatment with ATL III and decreased after overexpression of IGF2BP3, suggesting that ATL III has a regulatory effect on HeLa and SiHa cells that is mediated by IGF2BP3.

### 3.7 ATL III inhibits the binding of ETV5 to the transcription start site of IGF2BP3

**FIGURE 6:**
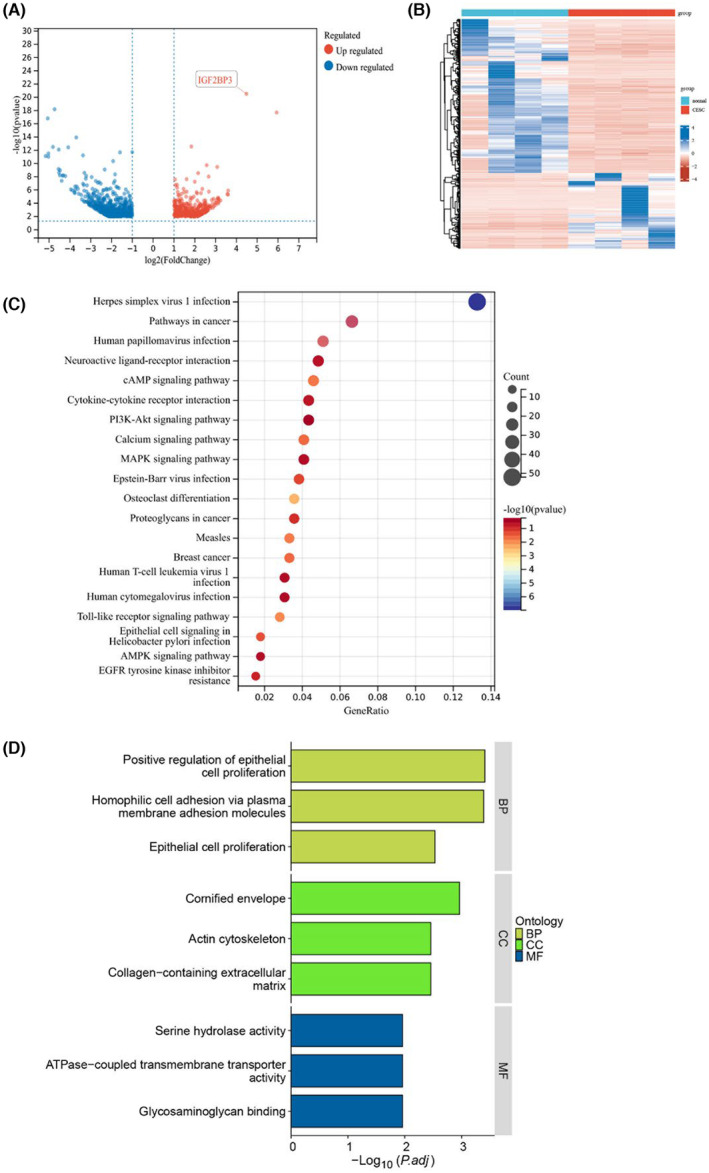
Bioinformatics analysis of DEGs in normal cervical tissue and cervical cancer tissue. (A and B) The volcano map and heatmap show upregulated and downregulated genes among the DEGs. (C and D) The enriched KEGG and GO functional pathways.

**FIGURE 7:**
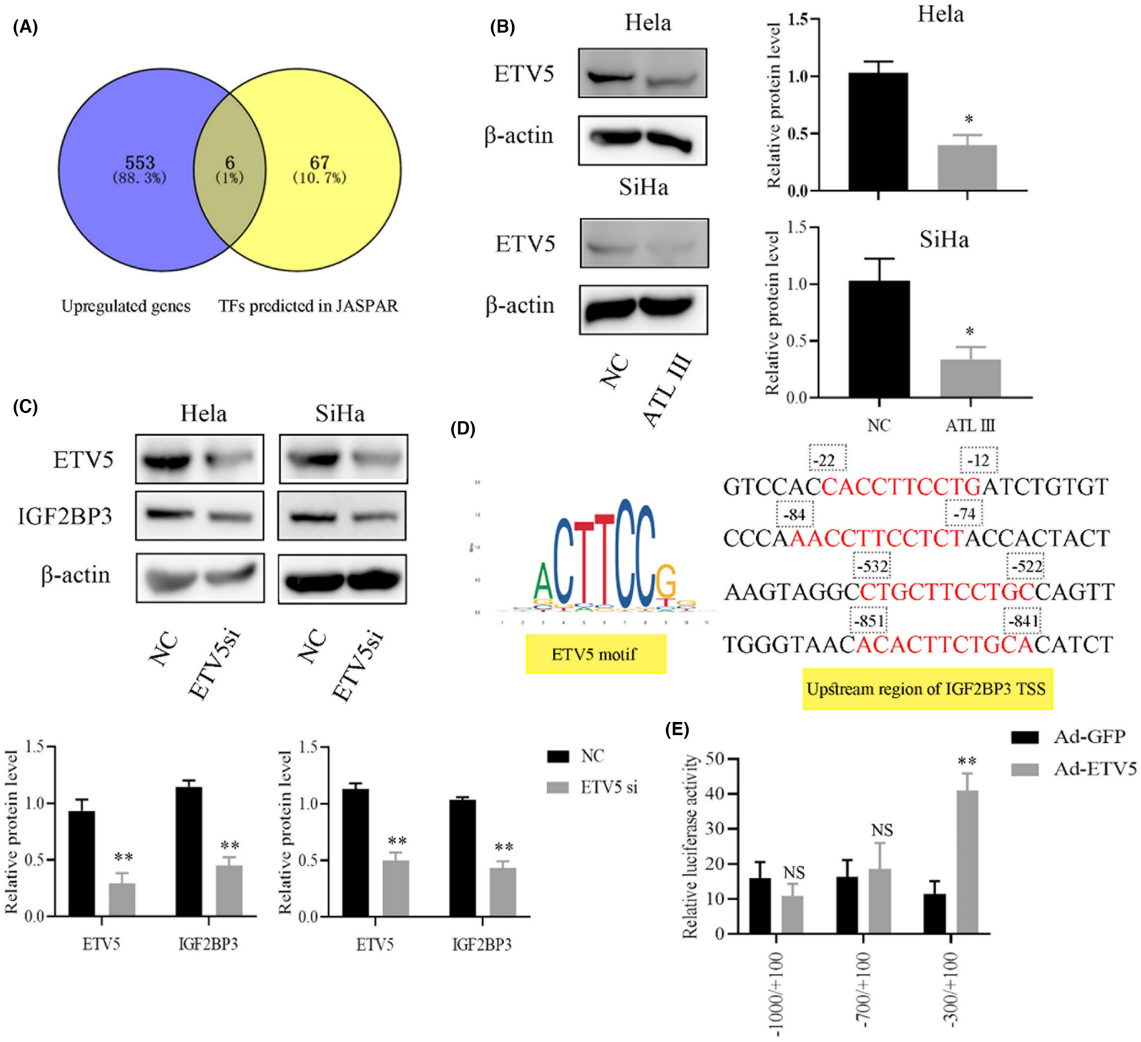
ATL III inhibits the binding of ETV5 to the transcription start site of IGF2BP3. (A) JASPAR was used to predict the potential transcription factor of IGF2BP3, which intersected with the upregulated gene in RNA‐Seq. (B) The protein expression of ETV in ALT III‐treated cells was detected. (C) Protein expression levels of IGF2BP3 and ETV5 were detected after the use of ETV5 siRNA. (D) Possible binding sites and motifs of the ETV and IGF2BP3 transcription initiation regions. (E) HEK 293 T cells were cotransfected with IGF2BP3 promoter plasmids (−1000/+100 bp, −700/+100 bp, and − 300/+100 bp) and ETV5 plasmids. Then, luciferase activity was measured. Data are presented as the mean ± SEM. *N* = 3. *Compared with NC *p* < 0.05, **compared with NC *p* < 0.01.

To further explore the mechanism of ALT III on IGF2BP3, we used JASPAR to predict possible transcription factors and obtained 73 potential transcription factors. Then, we crossed the genes with increased expression in the sequencing results of Figure 6 and obtained six transcription factors with high expression in tumour tissue (Figure 7A). We subsequently found that the expression level of ETV5 in cells treated with ALT III decreased significantly (Figure 7B). To evaluate whether ETV5 can regulate the level of IGF2BP3, we designed an ETV5 siRNA and found that the level of IGF2BP3 can also be reduced after artificially reducing the level of ETV5 (Figure 7C). This finding implies that ETV5 may be the upstream transcription factor for IGF2BP3. We speculated the possible binding sites and motifs of the ETV and IGF2BP3 transcription initiation regions (Figure 7D). Then, we generated plasmids expressing luciferase reporter genes driven by IGF2BP3 promoter fragments (−1000/+100 bp, −700/+100 bp, and − 300/+100 bp). The luciferase reporter gene results showed that ETV5 was bound to the upstream promoter of IGF2BP3, and the binding region was −300/+100 bp (Figure 7E).

## 4 DISCUSSION

ATL III is a compound with remarkable pharmacological activity that was isolated from *Atractylodes macrocephala* and has anticancer, anti‐inflammatory and antioxidative pharmacological effects. According to a recent study, ATL‐III has been shown to have antidepressant effects, as ATL‐III can protect PC12 cells from corticosterone‐induced damage by inhibiting intracellular Ca^2+^ overload, inhibiting mitochondrial apoptotic pathways and regulating the inflammatory pathways of MAPK/NF‐κB.^16^ ATL‐III has a significant inhibitory effect on DOI‐induced HTR, and its B‐C ring is similar to serotonin in structure, suggesting that the B‐C ring may be partially involved in the antagonistic activity against serotonin receptors, suggesting that ATL‐III has an antihallucinogenic effect.^17^ Many studies have also shown that ATL III inhibits the expression of caspase‐3, thereby inhibiting the apoptosis of neurons in the cerebral cortex of mice, and shows a strong neuroprotective effect.^18^, ^19^

In addition to its neuroprotective effect, ATL III also has a strong anti‐inflammatory effect. ATL‐III could inhibit the production of nitric oxide and PGE2 and induce the expression of nitric oxide synthetase and COX‐2 in LPS‐induced RAW 264.7 cells.^20^ ATL III also inhibits LPS‐induced TNF‐α and IL‐6 release in RAW264.7 cells by inhibiting the NF‐κB and MAPK signalling pathways.^21^ Although this preliminary study did not clarify the mechanism of action of ATL III in cervical cancer, the main innovation of this study is that the role of ATL III was explored in cervical cancer cells for the first time. The results show that ATL III has a strong effect on promoting apoptosis and inhibiting the migration and invasion of HeLa and SiHa cells.

Previous studies have reported that IGF2BP3 is highly expressed in various types of cancer. Many studies have shown that IGF2BP3 has a promoting role in the development of tumours. Accordingly, high expression of IGF2BP3 has been detected in some precancerous human diseases, such as abnormal hyperplasia in Barrett's oesophagus and tumours in the pancreas.^22^, ^23^ IGF2BP3 is a highly conserved gene with few mutations, and its stability is favourable for its use as a tumour marker. Studies have found that the transcription factor NF‐κB directly binds to the IGF2BP3 promoter region after entering the nucleus to maintain and migrate glioma stem‐like cells.^24^ IGF2BP3 is highly expressed in ovarian mucus tumours and is positively correlated with malignant tumours, indicating that IGF2BP3 can be used for the differential diagnosis and progression monitoring of ovarian tumours.^25^ The expression of IMP3 in CD44^+^CD24‐ESA^+^ cell clusters in breast cancer tissue was significantly upregulated. The expression level of IMP3 in mesenchymal cells was higher than that in epithelial cells.^26^ In leukaemia, IGF2BP3 helps stabilize COX‐2 mRNA and facilitates the translation of the key mediators of inflammatory and antiapoptotic signals in leukaemia cells.^27^

Moreover, many experiments have indicated that IGF2BP3 has a role in promoting or maintaining tumour cell subsets with stem cell characteristics and is a contributing factor in the establishment and progression of tumours. Vikesaa compared the phenotypes of IGF2BP3‐silenced cells and nonsilenced cells in vitro and observed that tumour cells expressing IGF2BP3 showed significant cell elongation and motility phenotypes, such as increased adhesion between cells.^6^ Zhao observed that overexpression of IGF2BP3 can promote cell proliferation, tumour migration and invasion in vitro, while low expression of IGF2BP3 has the opposite effect, and IGF2BP3 can promote tumour metastasis in vivo.^26^ In summary, multiple signalling pathways are involved in the promotion of tumour growth by IGF2BP3.

Currently, the mechanism of IGF2BP3 in cervical cancer is obscure. In our study, IGF2BP3 promoted the migration and invasion of HeLa and SiHa cells in vitro, indicating that IGF2BP3 plays a vital role in the invasion and metastasis of cervical cancer. After predicting the potential of IGF2BP3 transcription factors, we found several factors that were highly expressed in tumour tissue RNA‐Seq. We found that ETV5 may promote IGF2BP3 transcription by binding to the IGF2BP3 transcription start site within 300 bp, and the expression of ETV5 was also regulated by ALT III.

In this study, we first discovered that ATL III has a strong inhibitory effect on cervical cancer cells through treatment of HeLa (HPV18^+^) and SiHa (HPV16^+^) cervical cells, suggesting that ATL III has the potential to become a compound for treating cervical cancer. We studied the mechanism of ATL III in cervical cancer cells, and according to the qRT–PCR results, ATL III downregulates IGF2BP3 in HeLa and SiHa cells, which indicates that IGF2BP3 may be a common target of ATL III in HeLa and SiHa cells. In addition, the rescue experiment showed that ATL III regulates the EMT of cervical cancer cells by downregulating IGF2BP3. Unfortunately, the effect of ATL III on cervical cancer needs to be validated in more translational trials.

## 5 CONCLUSIONS

**FIGURE 8:**
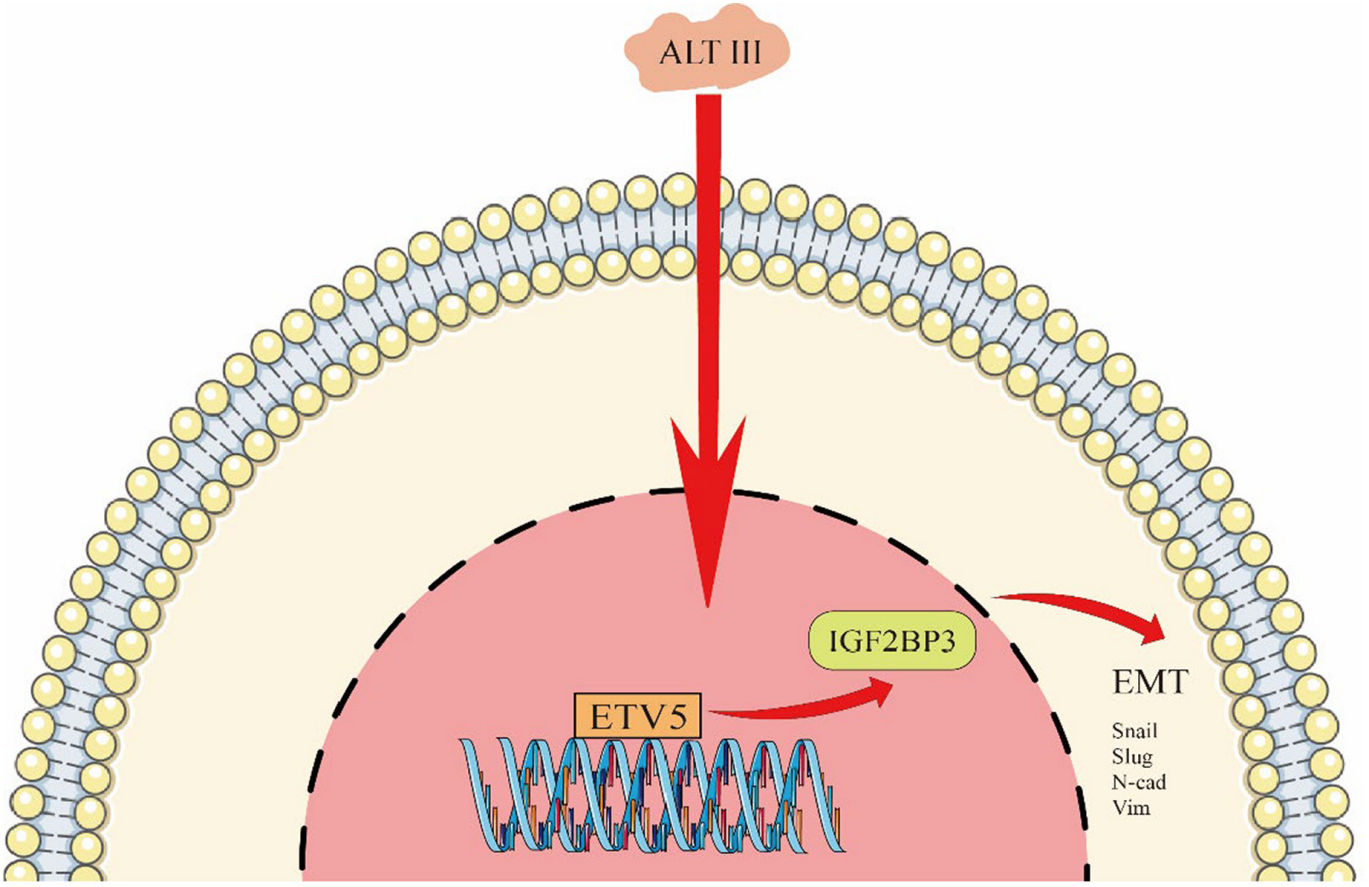
ALT III indirectly inhibits IGF2BP3 transcription by inhibiting the expression level of the transcription factor ETV5. Thus, Snail, Slug, N‐cad and Vim were reduced, which in turn suppressed EMT processes.

This study shows that ATL III can inhibit proliferation, migration and invasion in both HeLa and SiHa cells. Analysis of DEGs between normal cervical tissue and cancer tissue revealed IGF2BP3 as a common target gene for ATL III in both HeLa and SiHa cells. This study shows that ATL III can regulate EMT in cervical cancer cells and that overexpression of IGF2BP3 can reverse the inhibition of HeLa and SiHa cell proliferation. ETV5, as a transcription factor of IGF2BP3, can also be inhibited by ALT III (Figure 8). These results are of great significance, and ATL III or ALT III as lead compounds may provide potential programs to inhibit the recurrence and metastasis of cervical cancer.

## AUTHOR CONTRIBUTIONS

**Meixia Wang:** Conceptualization (equal); data curation (lead); formal analysis (lead); methodology (lead); visualization (lead); writing – original draft (lead). **Jingwen Meng:** Conceptualization (supporting); data curation (supporting); formal analysis (equal); methodology (equal); validation (equal); writing – original draft (supporting). **hongyun wang:** Formal analysis (supporting); investigation (supporting); methodology (supporting); resources (equal); software (supporting); visualization (supporting). **huijuan Hu:** Formal analysis (supporting); investigation (supporting); methodology (equal); validation (supporting); visualization (supporting); writing – original draft (supporting). **Ying Hong:** Conceptualization (equal); funding acquisition (lead); supervision (lead).

## FUNDING INFORMATION

This study was funded by the Scientific Research Project of the Institute of Science and Technology of the National Health Commission of the People's Republic of China (#2019HX017‐05) and China's 14th Five‐Year Plan, the major collaborative innovation project of the Medical and Health Science and Technology Innovation Project of the Chinese Academy of Medical Sciences (#2021‐I2M‐1‐004).

## CONFLICT OF INTEREST STATEMENT

No conflicts of interest, financial or otherwise, are declared by the authors.

## Supporting information


Data S1.

## Data Availability

The data that support the findings of this study are available from the corresponding author upon reasonable request.
