# Supplementary material for: Atractylodes macrocephala III suppresses EMT in cervical cancer by regulating IGF2BP3 through ETV5
Source: J Cell Mol Med. 2024 Feb 15;28(4):e18081. doi: 10.1111/jcmm.18081 (PMC10868144; doi:10.1111/jcmm.18081)
Supplement: Supplementary file 1 — Data S1. [file JCMM-28-e18081-s001.docx]

Table S1 Case information

Abbreviation:

CIN: Cervical intraepithelial neoplasias; CC: cervical cancer ; HPV: human papillomavirus; TCT: Thinprep cytologic test; Y: Yes; P: positive; N: negative; CIN I: cervical intraepithelial neoplasias I; CIN III: cervical intraepithelial neoplasias III; CSC: cervical squamous carcinoma; MUL: Multiple uterine Leiomyoma; AU: Adenomyosis of uterus;

| **No** | **Group** | **Age** | **BMI** | **Deliveries** | **Abortion** | **Menarche** | **Menopause** | **Condom use** | **Smoker** | **Hypertension** | **Diabetes** | **HPV** | **HPV infection history（Month）** | **TCT** | **Diagnose** |
| --- | --- | --- | --- | --- | --- | --- | --- | --- | --- | --- | --- | --- | --- | --- | --- |
| 1 | Normal | 47 | 28.4 | 1 | 1 | 12 |  |  |  |  |  | N | 0 | NILM | MUL |
| 2 | Normal | 44 | 32.3 | 1 | 0 | 13 |  | Y |  |  |  | N | 0 | NILM | MUL |
| 3 | Normal | 48 | 25.6 | 2 | 0 | 11 |  |  |  |  |  | N | 0 | NILM | 1、AU，2、MUL |
| 4 | Normal | 54 | 26.2 | 2 | 1 | 13 |  |  |  | Y | Y | N | 0 | NILM | AU |
| 5 | Normal | 44 | 23.4 | 1 | 2 | 12 |  | Y |  |  |  | N | 0 | NILM | MUL |
| 6 | Normal | 46 | 29.6 | 1 | 2 | 14 |  | Y | Y |  |  | N | 0 | NILM | MUL |
| 7 | Normal | 50 | 27.8 | 1 | 1 | 14 |  |  |  |  |  | N | 0 | NILM | AU |
| 8 | Normal | 48 | 25.8 | 2 | 0 | 13 |  |  |  |  |  | N | 0 | NILM | AU |
| 9 | Normal | 47 | 29.3 | 2 | 1 | 11 |  | Y |  |  |  | N | 0 | NILM | MUL |
| 10 | Normal | 45 | 30.1 | 1 | 1 | 11 |  | Y |  |  |  | N | 0 | NILM | MUL |
| 11 | Normal | 53 | 25.8 | 2 | 0 | 12 | Y |  |  |  |  | N | 0 | NILM | AU |
| 12 | Normal | 57 | 30.1 | 2 | 0 | 12 | Y |  |  | Y | Y | N | 0 | NILM | MUL |
| 13 | CIN I | 47 | 28.6 | 2 | 0 | 13 |  |  |  |  |  | P | 10 | NILM | CIN I |
| 14 | CIN I | 49 | 29.3 | 1 | 1 | 11 |  | Y |  |  |  | P | 6 | NILM | CIN I |
| 15 | CIN I | 58 | 30.1 | 3 | 0 | 12 | Y |  |  | Y |  | P | 10 | NILM | CIN I |
| 16 | CIN I | 32 | 27.5 | 0 | 2 | 13 |  | Y |  |  |  | P | 12 | NILM | CIN I |
| 17 | CIN I | 54 | 25.9 | 2 | 1 | 11 |  |  |  |  |  | P | 7 | NILM | CIN I |
| 18 | CIN I | 30 | 28.4 | 1 | 0 | 12 |  | Y | Y |  |  | P | 11 | NILM | CIN I |
| 19 | CIN I | 41 | 29.6 | 1 | 0 | 10 |  |  |  |  |  | P | 9 | NILM | CIN I |
| 20 | CIN I | 51 | 26.7 | 2 | 0 | 13 |  |  |  | Y | Y | P | 28 | ASCUS | CIN I |
| 21 | CIN I | 30 | 25.6 | 2 | 1 | 13 |  | Y |  |  |  | P | 18 | NILM | CIN I |
| 22 | CIN I | 31 | 27.1 | 0 | 2 | 11 |  |  |  |  |  | P | 12 | NILM | CIN I |
| 23 | CIN I | 44 | 29.5 | 1 | 0 | 15 |  |  |  |  |  | P | 10 | NILM | CIN I |
| 24 | CIN I | 27 | 22.3 | 0 | 2 | 12 |  | Y |  |  |  | P | 12 | NILM | CIN I |
| 25 | CIN I | 57 | 29.8 | 2 | 0 | 13 | Y |  |  | Y |  | P | 8 | NILM | CIN I |
| 26 | CIN I | 42 | 27.4 | 1 | 1 | 14 |  |  |  |  |  | P | 15 | NILM | CIN I |
| 27 | CIN III | 48 | 29.3 | 1 | 2 | 12 | Y | Y |  |  | Y | P | 18 | NILM | CIN III |
| 28 | CIN III | 30 | 25.3 | 1 | 0 | 11 |  |  |  |  |  | P | 12 | ASCUS | CIN III |
| 29 | CIN III | 50 | 29.4 | 2 | 2 | 12 |  |  |  | Y |  | P | 10 | NILM | CIN III |
| 30 | CIN III | 29 | 21.8 | 0 | 1 | 11 |  |  |  |  |  | P | 18 | ASCUS | CIN III |
| 31 | CIN III | 32 | 25.9 | 1 | 3 | 12 |  |  | Y |  |  | P | 20 | ASCUS | CIN III |
| 32 | CIN III | 52 | 32.8 | 2 | 1 | 13 | Y | Y |  |  |  | P | 18 | HSIL | CIN III |
| 33 | CIN III | 26 | 26.8 | 1 | 2 | 12 |  |  |  |  |  | P | 10 | NILM | CIN III |
| 34 | CIN III | 47 | 28.7 | 1 | 3 | 13 |  |  |  |  |  | P | 8 | HSIL | CIN III |
| 35 | CIN III | 58 | 30.9 | 2 | 0 | 14 | Y |  |  | Y | Y | P | 13 | ASCUS | CIN III |
| 36 | CIN III | 36 | 29.6 | 1 | 1 | 13 |  |  |  |  |  | P | 17 | HSIL | CIN III |
| 37 | CIN III | 42 | 33.8 | 1 | 2 | 13 |  |  |  |  |  | P | 14 | NILM | CIN III |
| 38 | CIN III | 40 | 27.5 | 2 | 1 | 11 |  | Y | Y |  |  | P | 12 | HSIL | CIN III |
| 39 | Cancer | 39 | 24.6 | 1 | 2 | 12 |  | Y |  |  |  | P | 18 | HSIL | CSC(Ib3) |
| 40 | Cancer | 68 | 29.2 | 2 | 2 | 14 | Y |  |  |  |  | P | 9 | HSIL | CSC(Ib2) |
| 41 | Cancer | 66 | 32.7 | 2 | 2 | 15 | Y |  |  | Y |  | P | 12 | HSIL | CSC(Ib3) |
| 42 | Cancer | 50 | 28.5 | 1 | 3 | 13 | Y |  |  |  |  | P | 3 | ASCUS | CSC(Ib1) |
| 43 | Cancer | 44 | 30.6 | 1 | 2 | 12 |  |  |  |  |  | P | 2 | ASCUS | CSC(Ib1) |
| 44 | Cancer | 43 | 27.8 | 1 | 1 | 12 |  | Y |  |  |  | P | 6 | HSIL | CSC(Ib1) |
| 45 | Cancer | 66 | 29.6 | 3 | 0 | 15 | Y |  |  | Y | Y | N |  | HSIL | CSC(Ib1) |
| 46 | Cancer | 38 | 25.1 | 2 | 2 | 11 |  | Y | Y |  |  | P | 4 | CC | CSC(Ib3) |
| 47 | Cancer | 46 | 27.4 | 2 | 1 | 13 |  |  |  |  |  | P | 3 | HSIL | CSC(Ib3) |
| 48 | Cancer | 68 | 25.9 | 1 | 2 | 14 | Y |  |  |  | Y | P | 1 | CC | CSC(Ib2) |
| 49 | Cancer | 66 | 28.2 | 2 | 0 | 13 | Y | Y |  |  |  | P | 7 | HSIL | CSC(Ib2) |
| 50 | Cancer | 53 | 31.7 | 2 | 2 | 12 |  |  |  | Y |  | P | 5 | HSIL | CSC(Ib1) |
| 51 | Paracancerous | 57 | 29.7 | 1 | 1 | 13 | Y |  |  |  |  | N |  | HSIL | CSC(Ib3) |
| 52 | Paracancerous | 47 | 27.3 | 1 | 3 | 11 |  |  |  |  |  | P | 15 | HSIL | CSC(Ib3) |
| 53 | Paracancerous | 56 | 30.2 | 2 | 0 | 14 | Y |  |  |  |  | P | 9 | HSIL | CSC(Ib2) |
| 54 | Paracancerous | 45 | 28.6 | 1 | 2 | 12 |  | Y |  |  |  | P | 4 | CC | CSC(Ib3) |
| 55 | Paracancerous | 59 | 26.9 | 2 | 2 | 14 | Y |  |  | Y |  | P | 6 | HSIL | CSC(Ib2) |
| 56 | Paracancerous | 39 | 26.9 | 1 | 4 | 12 |  | Y |  |  |  | N |  | HSIL | CSC(Ib3) |
| 57 | Paracancerous | 51 | 31.2 | 2 | 2 | 12 |  |  |  |  |  | P | 2 | CC | CSC(Ib3) |
| 58 | Paracancerous | 48 | 27.5 | 2 | 2 | 14 |  | Y | Y |  |  | P | 3 | CC | CSC(Ib2) |
| 59 | Paracancerous | 62 | 30.3 | 3 | 0 | 15 | Y |  |  |  | Y | P | 9 | HSIL | CSC(Ib3) |
| 60 | Paracancerous | 66 | 25.6 | 3 | 0 | 14 | Y |  |  | Y |  | P | 5 | CC | CSC(Ib3) |
| 61 | Paracancerous | 57 | 29.5 | 2 | 1 | 11 | Y |  |  |  |  | P | 4 | HSIL | CSC(Ib3) |
| 62 | Paracancerous | 62 | 31.1 | 2 | 2 | 12 | Y |  |  |  | Y | P | 3 | HSIL | CSC(Ib3) |

Table S2. Sequences of primer

| Name | Forward Primer | Reverse Primer |
| --- | --- | --- |
| ZIC5 | GTCTATGGGCCTGATTGTGTAGT | GCCAAATCCGCTAATCTCAGC |
| IGF2BP3 | TATATCGGAAACCTCAGCGAGA | GGACCGAGTGCTCAACTTCT |
| CXCL9 | CCAGTAGTGAGAAAGGGTCGC | AGGGCTTGGGGCAAATTGTT |
| CXCL10 | GTGGCATTCAAGGAGTACCTC | TGATGGCCTTCGATTCTGGATT |
| TNNT1 | TGATCCCGCCAAAGATCCC | TCTTCCGCTGCTCGAAATGTA |
| CRMP1 | AGTGACCGACTCCTCATCAAA | CCAGGAACGATTAAGTTCTCTCC |
| RGS4 | ACATCGGCTAGGTTTCCTGC | GTTGTGGGAAGAATTGTGTTCAC |
| TNFRSF11B | GCGCTCGTGTTTCTGGACA | AGTATAGACACTCGTCACTGGTG |
| CXCL11 | GACGCTGTCTTTGCATAGGC | GGATTTAGGCATCGTTGTCCTTT |
| SOX2 | GCCGAGTGGAAACTTTTGTCG | GGCAGCGTGTACTTATCCTTCT |
| MMP12 | CATGAACCGTGAGGATGTTGA | GCATGGGCTAGGATTCCACC |
| RNF157 | TGGACATCCCGTCTAATTCCG | CCTCCCATAATGAAGTGGCTG |
| GPRIN1 | AAAGCAGGCCGATTCCACTTC | TCCTTCCTCGGTGACACTGTA |
| ATP1A3 | AAGGAGGTGGCTATGACAGAG | GTGAGTGCGTTAGGCCCAT |
| 33643 | CCACGGTCACACTGTCTACT | GACGTTGCAGTTGTGGTTCT |
| ETV5 | TCAGCAAGTCCCTTTTATGGTC | GCTCTTCAGAATCGTGAGCCA |
| 18S | GGCCGTTCTTAGTTGGTGGAGCG | CTGAACGCCACTTGTCCCTC |
| GAPDH | GGAGCGAGATCCCTCCAAAAT | GGAGCGAGATCCCTCCAAAAT |
